# Emotion appraisal dimensions inferred from vocal expressions are consistent across cultures: a comparison between Australia and India

**DOI:** 10.1098/rsos.170912

**Published:** 2017-11-15

**Authors:** Henrik Nordström, Petri Laukka, Nutankumar S. Thingujam, Emery Schubert, Hillary Anger Elfenbein

**Affiliations:** 1 Department of Psychology, Stockholm University, Stockholm 10691, Sweden; 2 Department of Psychology, Sikkim University, Gangtok 737102, India; 3 School of the Arts and Media, University of New South Wales, Sydney, New South Wales 2052, Australia; 4 Olin Business School, Washington University in St Louis, St Louis, MO 63130, USA

**Keywords:** acoustic parameters, appraisal, cross-cultural, emotion recognition, speech, vocal expression

## Abstract

This study explored the perception of emotion appraisal dimensions on the basis of speech prosody in a cross-cultural setting. Professional actors from Australia and India vocally portrayed different emotions (anger, fear, happiness, pride, relief, sadness, serenity and shame) by enacting emotion-eliciting situations. In a balanced design, participants from Australia and India then inferred aspects of the emotion-eliciting situation from the vocal expressions, described in terms of appraisal dimensions (novelty, intrinsic pleasantness, goal conduciveness, urgency, power and norm compatibility). Bayesian analyses showed that the perceived appraisal profiles for the vocally expressed emotions were generally consistent with predictions based on appraisal theories. Few group differences emerged, which suggests that the perceived appraisal profiles are largely universal. However, some differences between Australian and Indian participants were also evident, mainly for ratings of norm compatibility. The appraisal ratings were further correlated with a variety of acoustic measures in exploratory analyses, and inspection of the acoustic profiles suggested similarity across groups. In summary, results showed that listeners may infer several aspects of emotion-eliciting situations from the non-verbal aspects of a speaker's voice. These appraisal inferences also seem to be relatively independent of the cultural background of the listener and the speaker.

## 1. Introduction

The human voice is a versatile channel for emotional communication. Whenever we hear an individual speak, we do not only process the meaning of the words that are used, but also draw inferences based on the non-verbal aspects of the individual's voice. These inferences are not restricted to emotion categories such as ‘happy’ and ‘sad’, but can also include aspects of the situation that elicited the emotion [1]. The current article presents the first study of cross-cultural similarities and differences with regard to judgements of emotion-eliciting situations, described in terms of appraisal dimensions, from emotional speech prosody.

The majority of previous research on vocal expressions, and emotion expressions in general, has focused on the perception of emotion categories (for a review, see [2]). In a typical study, participants are presented with stimuli that are intended to express various emotion categories, and are then asked to identify the expressed emotion by selecting the appropriate label from a list of fixed options. Meta-analyses of vocal expression studies in this tradition have shown that categories such as anger, fear, happiness and sadness are generally recognized with accuracy rates well above chance both within and across cultures [3,4]. Several emotion categories are also associated with relatively distinct patterns of acoustic characteristics (e.g. [4]). Although emotion categories can be well recognized across cultural groups, results from cross-cultural studies often also report evidence for in-group advantage, to the effect that emotion recognition is more accurate when speakers and perceivers come from the same culture versus from different cultures (e.g. [5–8]). Recently, it has been proposed that in-group advantage results from a greater match between expression and perception styles in conditions where speakers and perceivers are sampled from the same cultural group [9,10].

However, the use of emotion categories has been criticized on the ground that such labels may ‘lack clarity with regard to whether one, some, or all emotion components are perceived’ [11, p. 47]. Emotions are commonly viewed as consisting of several components, including cognitive appraisals, peripheral physiological responses, action tendencies, expressive and instrumental behaviour, and subjective feelings (e.g. [12]). From an appraisal perspective, the response is initiated by largely automatic evaluations of the significance of a situation or event, which then drive the responses in the other components in a dynamic process. Appraisal theories suggest that such evaluations are conducted along a number of dimensions related to relevance, implications, coping and normative significance (e.g. [12,13]). Scherer [14] proposed that vocal expressions may reflect the antecedent cognitive appraisal processes that produced the emotional response in the speaker, and went on to hypothesize that this ‘should allow the listener to reconstruct the major features of the emotion-producing event in its effect on the speaker’ [15, p. 94]. Testing this hypothesis, Laukka & Elfenbein [1] showed that listeners were able to consistently infer several aspects of emotion-eliciting situations described in terms of appraisal dimensions. Recent studies have also reported that appraisal dimensions may be perceived from other modalities of expression such as facial expressions (e.g. [11,16,17]), which suggests that appraisal information is not limited to vocal expressions.

The current article extends previous cross-cultural research on vocal expression by investigating how listeners from Australia and India perceive appraisal dimensions from vocal expressions recorded by speakers from the same two nations in a balanced design. We chose to compare Australia and India because these two nations exhibit different profiles on Hofstede's [18] cultural dimensions. Australia scores low on power distance and high on individualism, whereas the opposite pattern is reported for India. In a previous study, using a categorical approach, we also observed a clear bi-directional in-group advantage, with more accurate emotion recognition accuracy in in-group versus out-group conditions, between these two nations [10]. Except for the novel cross-cultural component, we employed a similar method as previously used in Laukka & Elfenbein [1]. Professional actors from Australia and India were instructed to vocally express a wide range of emotions by enacting emotion-eliciting situations. The resulting expressions were then rated by individuals from both nations with respect to appraisal dimensions, which represent our cognitive understanding of the situations that elicit emotions.

We used a Bayesian data analytic strategy to answer the following two research questions. First, we investigated whether appraisal ratings for the different emotions were consistent with predictions based on appraisal theory. Second, we investigated the effects of culture by testing if there were differences between Australian and Indian participants' appraisal rating scores. The answers to these questions are of importance both for our conceptual understanding about the type of information that can be conveyed by vocal expressions and for our understanding of universality and cultural specificity in emotion perception.

Finally, we investigated the extent to which listeners’ appraisal ratings were associated with various acoustic parameters in exploratory analyses. Laukka & Elfenbein [1] reported correlations between appraisal ratings and acoustic parameters in a within-cultural setting, but no previous study has investigated such correlations in a cross-cultural setting. These analyses thus provide initial clues about cultural similarities and differences in the degree to which various acoustic parameters are used by listeners to infer appraisal information from emotional speech prosody.

## 2. Material and methods

### 2.1. Participants

Ninety individuals from Australia (53 women; mean age = 21.0 years, s.d. = 3.8, range = 18–46 years) and 40 Indian individuals (23 women; mean age = 22.1, s.d. = 2.1, range = 18–28 years) took part in the experiment. All participants reported being born and raised in their respective country. Australian participants were recruited at the University of New South Wales in Sydney, Australia, and participated as part of their course requirements. Indian participants were students at Sikkim University in Gangtok, India, and received a cash reward (INR 300) for their participation. All Australian participants reported English as their primary language, and the most common primary languages in the Indian participant group were Nepali, Assamese, Hindi and Tibetan. None of the Indian participants had English as their primary language, but all reported being fluent in English and regularly used the language as students at a university where the medium of instruction is English.

Because the Australian participants were students at a highly multicultural university, data were also recorded from 71 additional students who were not born in Australia and did not have English as their native language. These data are not included in the present study.

### 2.2. Stimulus material and acoustic analyses

Audio recordings of vocal emotion expressions were obtained from the cross-cultural VENEC database [10], which contains emotion portrayals from several English-speaking cultures including Australia and India. Professional actors from Australia and India (*N* = 20 per culture, 50% women) were instructed to enact scenarios commonly associated with the emotions anger, fear, happiness, pride, relief, sadness, serenity and shame. The scenarios described typical situations in which each emotion may be elicited according to current emotion research (e.g. [13,19,20]). For consistency across portrayals, in each case, the verbal content consisted of brief emotionally neutral sentences in English (‘Let me tell you something’, ‘That is exactly what happened’). See Laukka *et al*. [10] for a full description of the recording procedure including descriptions of the emotion scenarios.

We selected eight recordings from each of the eight emotion categories/scenarios from both the Australian and Indian recordings for the current study, which resulted in a total of 128 vocal expressions. The selected portrayals contained stimuli from 19 Australian (nine female) and 19 Indian (10 female) actors, with the following numbers of female (Australia, *N* = 32; India, *N* = 37) and male (Australia, *N* = 32; India, *N* = 27) stimuli. Stimulus selection was based on own-culture emotion recognition rates obtained in [10], using an 11-alternative forced-choice paradigm conducted separately for positive and negative emotions. The average recognition rate for the selected portrayals was around 4.5 times higher than chance agreement (mean recognition accuracy = 41%; Australia = 35%, India = 47%).

For the current study, all Australian and Indian emotion portrayals in the VENEC database were acoustically analysed using the *openSMILE* software [21] to extract the parameters included in the Geneva Minimalistic Acoustic Parameter Set (GeMAPS; see [22] for a detailed description). The GeMAPS was recently proposed as a standardized baseline set of acoustic cues for research on affective speech, and contains cues related to frequency, energy, spectral balance and temporal features of the voice. We used the extended parameter set which includes 88 acoustic cues, as described in Eyben *et al*. [22]. The raw values for each cue and speaker were *z*-transformed, to control for individual differences in baseline values between speakers, before inclusion in the statistical analyses (e.g. [23,24]).

To reduce the number of acoustic parameters in the subsequent analyses, we performed a principal component analysis (PCA) with varimax normalized rotation on the selected set of 128 vocal portrayals. Parallel analysis was used to indicate the number of factors to retain, using the ‘paran’ package in R [25,26], and revealed a nine-factor solution. Based on the PCA, the cues with the highest loadings and/or interpretability were chosen to represent each factor. The selected cues (*N* = 11) and their factor loadings are shown in table 1, which also includes a brief description of each cue.

**Table 1.** Summary of selected acoustic parameters.

feature type	description	factor loading
*frequency cues*		
F0M	mean fundamental frequency (F0) on a semitone frequency scale	factor 4: 0.76
F0SD	variability (standard deviation) of F0	factor 3: 0.73
F1FreqM	mean of first formant (F1) centre frequency	factor 6: 0.76
*energy cues*		
IntM	mean voice intensity estimated from an auditory spectrum	factor 1: 0.90
IntSD	variability (standard deviation) of voice intensity	factor 5: 0.87
*spectral balance cues*		
F1amplitude	relative energy of the spectral envelope in the first formant region	factor 2: −0.85
Hammarberg	Hammarberg index, i.e. the ratio of the strongest energy peaks in the 0–2 versus 2–5 kHz regions	factor 7: 0.83
spectral slope	mean spectral slope (i.e. linear regression slope of the logarithmic power spectrum) for the 500–1500 Hz region	factor 9: 0.73
spectral flux *M*	mean spectral flux, i.e. the difference between the spectra of two consecutive speech frames	factor 1: 0.89
spectral flux s.d.	variability (standard deviation) of spectral flux	factor 5: 0.88
*temporal cues*		
VoicedSegPerSec	the number of continuous voiced regions per second (pseudo syllable rate)	factor 8: −0.83

### 2.3. Procedure

Experiments were conducted individually and *PsychoPy* software [27] was used to present stimuli and register responses. Participants listened to the stimuli through headphones with constant sound levels and two practice rounds preceded the experiment proper. On-screen instructions informed participants that they should try to imagine what type of event the speaker in the recording was reacting to, and that they should answer the following six questions about the imagined event associated with each recording: ‘Did the event occur suddenly and abruptly?’ (Novelty), ‘Was the event pleasant?’ (Pleasantness), ‘Did the event help the speaker to reach a goal or satisfy a need?’ (Goal conduciveness), ‘Did the event require the speaker to respond urgently?’ (Urgency), ‘Could the outcome of the event be modified by the speaker's actions?’ (Power) and ‘Was the event compatible with the speaker's norms?’ (Norm compatibility). All questions were presented simultaneously on the screen, but the order of the questions was randomized within sessions. The scale ranged from 1 (No, not at all) to 5 (Yes, absolutely) for all appraisal dimensions.

Stimulus presentation order was randomized within sessions. Participants were allowed to play the same recording as many times as needed to reach a decision, and the next recording started automatically when the participants had finished all six appraisal ratings. In Australia, the experiment was timed to last 30 min regardless of how many stimuli had been rated. Ten Australian participants did not rate stimuli from all emotion categories from both speaker cultures and were therefore excluded in the analysis. The remaining 80 participants rated on average 82.3 (s.d. = 23.6, range 43–128) stimuli, and at least one stimulus from each emotion category (*M* = 5.1, s.d. = 1.9). This resulted in at least 40 Australian ratings per stimulus (*M* = 51.4, s.d. = 4.3, max = 63). In India, all participants instead rated the complete set of 128 recordings resulting in 40 Indian ratings per stimulus.

### 2.4. Appraisal predictions

Following Laukka & Elfenbein [1], and based on the literature on emotion appraisal (e.g. [13,28]), we set up the following predictions about the expected outcome of the participants' appraisal ratings. All appraisal dimensions that received acceptable inter-rater reliability in [1] were also included in the current study. Note that it was not possible to make predictions for all appraisal dimensions and all emotions based on the literature.

(a) Novelty: high = anger, fear, happiness; low = sadness, serenity
(b) Pleasantness: high = happiness, pride, relief, serenity; low = anger, fear, sadness, shame
(c) Goal conduciveness: high = happiness, pride, relief; low = anger, fear, sadness, shame
(d) Urgency: high = anger, fear; low = happiness, relief, sadness, serenity
(e) Power: high = anger, happiness, pride; low = fear, sadness, shame
(f) Norm compatibility: high = happiness, pride; low = anger, shame

### 2.5. Statistical analyses

All statistical analyses were conducted using R [29] and all data and analysis code is available in the electronic supplementary material.

#### 2.5.1. Bayesian analyses

Bayesian analysis is one of many statistical tools, each with its benefits and drawbacks. In the current study, we have used Bayesian analyses because we believe that it gives the most accurate and informative description of our data. For example, its ability to differentiate between a small effect and low power [30] allowed us to interpret both similarities and differences found across cultures.

**Figure 1.**
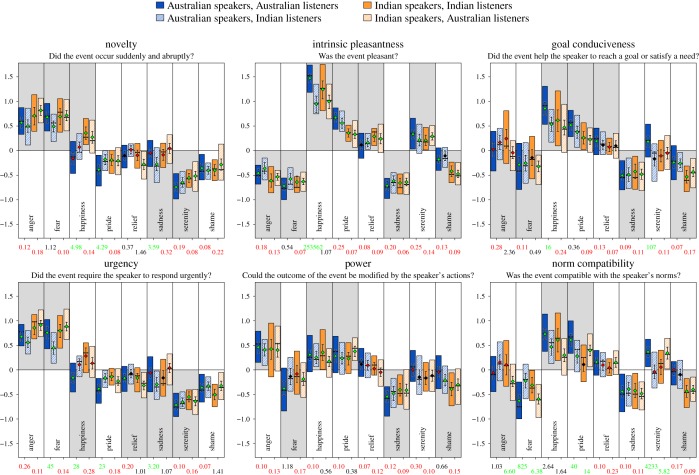
Participants' mean ratings (and boxplots) of appraisal dimensions (*z* scores) as a function of culture and the intended emotion of the vocal expressions. Colour of the boxes indicates speaker and perceiver culture: solid blue boxes, Australian stimuli, Australian participants; striped blue boxes, Australian stimuli, Indian participants; solid orange boxes, Indian stimuli, Indian participants; and striped orange boxes, Indian stimuli, Australian participants. Error bars indicate 95% CIs and shaded areas indicate direction of theoretical predictions. Diamonds indicate the mean appraisal ratings and the colour of the diamonds indicates if the associated Bayes factor (BF) supports the prediction (green, BF > 3), supports a population mean close to zero (red, BF < 1/3) or if both hypotheses are equally likely (black, 3 > BF > 1/3). Numbers presented on the *y*-axis show BFs for comparisons of ratings between Australian and Indian participants, and the colours indicate if the BF supports a difference between participant cultures (green, BF > 3), supports no difference between cultures (red, BF < 1/3) or if both hypotheses are equally likely (black, 3 > BF > 1/3).

To control for individual differences in baseline values between participants and rating scales, the raw rating values for each appraisal dimension and participant were *z*-transformed. The *z*-transformed ratings were then used to calculate an average rating for each participant across stimuli belonging to the same emotion category for each appraisal dimension and speaker culture. Participant averages were then used to calculate means and standard errors (s.e.) across participants from the same culture. This procedure resulted in 192 mean and associated s.e. values: *six* appraisal dimensions, *eight* emotion categories and *four* cultural conditions (in-group and out-group judgements for both participant groups), see the legend in figure 1.

To investigate the first research question (i.e. if ratings are consistent with predictions based on appraisal theory), we calculated the prior, likelihood and posterior probability distributions for a range of plausible mean values (−3 to 3). For appraisal dimensions and emotions for which it was possible to make predictions from the literature, the prior probability was defined as a half normal distribution in the direction of the prediction with a standard deviation of 1. To exemplify, the literature predicts that participants should assign high novelty ratings to expressions of anger. A half normal distribution describes this prediction well by declaring that, if the prediction is true, a mean value below 0 is very unlikely, a mean value above but close to 0 is most likely and a mean value above 0 is decreasingly unlikely as it becomes more extreme.

For appraisal dimensions and emotions for which it was *not* possible to make predictions from the literature, the prior probability was defined as a normal distribution centred on 0 with a standard deviation of 1. This distribution declares that a mean value close to 0 is most likely and that extreme mean values in either direction are increasingly unlikely. The likelihood was defined as a normal distribution centred on the obtained mean value with a standard deviation of the obtained s.e. value. The posterior probability distribution was calculated by multiplying the prior distribution with the likelihood distribution. The prior, likelihood and posterior distributions were standardized to have the same area under the curve.

To facilitate interpretation of the posterior probability distribution, a credible interval (CI) defined as the narrowest range covering 95% of the posterior probability distribution was calculated (see [30,31], and the R-script in the electronic supplementary material for a detailed description of the calculations). To further facilitate interpretation of the results, the Savage–Dickey density ratio method [32] was used to calculate a Bayes factor (BF) defined as a ratio of the heights of the prior and posterior distributions at the point where they intersect with zero. If this BF is larger than 1, it can be interpreted as support for an effect in the predicted direction rather than a population mean close to zero.

As a joint estimate of the accuracy of the predictions, a posterior probability was calculated using the beta function with the number of predictions (+1) and the number of BFs that were smaller than 3 (+1) as shape 1 and shape 2, respectively [31,33]. Three is an arbitrary cut-off, but it has been suggested to give ‘substantial’ evidence for an effect in the predicted direction [34]. Posterior probability distributions of the accuracy of the predictions split into in-group and out-group ratings for each listener culture were calculated the same way as for all predictions.

To investigate the second research question (i.e. if there are differences between Australian and Indian rating scores), prior, likelihood and posterior probability distributions were calculated for a range of plausible mean differences between participant cultures (−3 to 3). Because we had no predictions about the difference between cultures, the prior was always defined as a normal distribution centred on 0 with a standard deviation of 1. The likelihood was defined as a normal distribution centred on the obtained mean difference between participant cultures with a standard deviation of the obtained pooled s.e. of the mean difference. The posterior distribution and a BF were calculated the same way as described above. If this BF is larger than 1, it can be interpreted as support for a difference between Australian and Indian rating scores in the direction of the obtained data rather than a population mean difference close to zero.

#### 2.5.2. Acoustic correlates

To investigate the third research question (i.e. to investigate the acoustical correlates of appraisal ratings across cultures), average rating scores for each vocal stimulus were calculated for each combination of appraisal dimension and listener group. The average rating scores were then correlated (Pearson *r*) with the *z*-transformed values of the selected acoustic cues shown in table 1. This research question was exploratory and was therefore evaluated using descriptive rather than inferential statistics.

#### 2.5.3. Inter-rater reliability

Consistency among raters was assessed separately for each appraisal dimension and rater group (Australia and India). Raters were randomly split into halves and 10 000 randomly selected split-half correlations were calculated. The means of these correlations were then standardized using the Spearman–Brown prophecy formula [35]. As a validation of this procedure, we also calculated intra-class correlations, ICC(2,*k*), for the Indian raters using the ‘psych’ package in R [36]. Both procedures yielded almost (to the second decimal point) identical results. Because a majority of the Australian listeners did not rate all stimuli, it was not possible to calculate intra-class correlations for the Australian listeners. Therefore, the Spearman–Brown standardized mean split-half correlations were used as a measure of inter-rater reliability for both rater groups.

## 3. Results

A graphic summary of the results is shown in figure 1. The boxes in figure 1 show the first, second (median) and third quartiles for each appraisal dimension and emotion for each combination of listener and speaker culture. Blue boxes denote that speakers were Australian and orange boxes denote that speakers were Indian. Filled boxes denote that listeners were from the *same* culture as the speakers, and striped boxes denote that listeners were from a different culture than the speakers (see legend, figure 1, for more details). Finally, the grey background areas denote the direction of the predictions based on appraisal theory. Below, we detail how the data shown in figure 1 were used to answer the first two research questions. Statistics from all analyses, including BFs, are shown in the electronic supplementary material, table S1.

Inter-rater reliability (average split-half correlations) was good for all appraisal dimensions across cultural conditions. For Australian and Indian listeners, respectively, the inter-rater reliability for each dimension was: novelty (0.93, 0.95), intrinsic pleasantness (0.97, 0.98), goal conduciveness (0.88, 0.92), urgency (0.94, 0.96), power (0.82, 0.88) and norm compatibility (0.83, 0.93).

### 3.1. Are ratings consistent with predictions based on appraisal theory?

The boxes in figure 1 can be used to get a notion of the accuracy of the predictions. Boxes in grey areas that do not overlap zero indicate that at least 75% of the participants from that listener culture made ratings in the predicted direction. The green, red and black diamonds superimposed on the boxes in figure 1 show mean ratings for each listener culture, and the error bars adjacent to the diamonds show 95% CIs of the mean ratings. The colour of the diamonds indicates if the associated BF (described in the Material and methods section) supports the prediction (green, BF > 3) or a population mean close to zero (red, BF < 1/3), or if both hypotheses are equally likely (black, 3 > BF > 1/3).

Results showed that the perceived appraisal profiles for the different emotions were generally consistent with predictions. Counting the green diamonds in grey areas showed that 113 out of 144 (78%) mean ratings were in the predicted direction. For the remaining 31 predictions, 21 (15%) gave support for a mean close to zero and 10 (7%) did not support one hypothesis over the other. The posterior probability distributions of the joint accuracy of the predictions are shown in the electronic supplementary material, figure S1. A figure showing appraisal ratings for each emotion collapsed across cultures is also available in the electronic supplementary material, figure S2.

A comparison of the number of supported predictions between in-group and out-group conditions further showed that there was no imbalance between conditions. Out of the 113 supported predictions, 28 and 27 were from Australian and Indian participants in in-group conditions, and 27 and 31 were from Australian and Indian participants in out-group conditions.

### 3.2. Are there differences between ratings from Australian and Indian listeners?

The numbers presented on the horizontal axis in figure 1 show BFs (described in the Material and methods section) for each comparison of ratings between Australian and Indian participants. To facilitate interpretation, the same colour-coding as for the mean ratings was used. Green (BF > 3) indicates that the BF may be interpreted as support for a difference between participant cultures, red (BF < 1/3) as support for no difference and black (3 > BF > 1/3) that both hypotheses are equally likely.

Overall, the results indicated that appraisal ratings were relatively consistent across cultures. Out of the 96 comparisons, 17 (18%) of the BFs gave support for a difference between listener cultures, 61 (64%) gave support for no difference and 18 (19%) did not support one hypothesis over the other. The largest number of cultural differences were found for ratings of norm compatibility across emotions (figure 1), although we note that, with only 16 cross-cultural comparisons for each dimension, the estimates for individual appraisal dimensions are relatively uncertain. The posterior probability distributions of the proportion of BFs that supported no difference between listener groups are shown in the electronic supplementary material, figure S3.

### 3.3. Acoustical correlates of appraisal dimensions across cultures

Table 2 shows the correlations between ratings of each appraisal dimension and the selected acoustic cues for each combination of speaker and listener culture. These correlations give indications of which acoustic cues were used by the listeners to make inferences about the emotion antecedent situations. We focus on the associations that showed the largest effects (*r* > approx. 0.30) to assess the cross-cultural consistency of the acoustical correlates of appraisal dimensions and summarize the major trends below.

**Table 2.** Correlations (Pearson's *r*) between selected acoustic parameters and participants’ mean ratings of appraisal dimensions for each combination of listener and speaker culture. Note: *N* = 64. Bold type indicates *r* ≥ 0.30.

		novelty		urgency		intrinsic pleasantness		goal conduciveness		norm compatibility		power	
acoustic cue	listener culture speaker culture	Aus	Ind	Aus	Ind	Aus	Ind	Aus	Ind	Aus	Ind	Aus	Ind
*frequency cues*													
F0M	Aus	**0**.**46**	**0**.**45**	**0**.**50**	**0**.**45**	−0.05	−0.11	−0.03	0.02	−0.18	−0.09	−0.12	0.04
	Ind	**0**.**56**	**0**.**51**	**0**.**55**	**0**.**47**	−0.26	−0.15	−0.18	0.08	−**0**.**39**	0.00	−0.06	0.09
F0SD	Aus	−0.25	−0.24	−0.27	−**0**.**32**	−0.13	−0.10	−0.13	−0.22	−0.10	−0.19	−0.12	−0.24
	Ind	0.25	0.15	0.20	0.11	−0.27	−0.21	−0.23	−0.11	−**0**.**36**	−0.11	−0.15	−0.05
F1FreqM	Aus	0.21	0.23	0.20	0.16	−0.10	−0.05	0.01	0.02	−0.16	−0.01	−0.12	0.02
	Ind	**0**.**56**	**0**.**54**	**0**.**55**	**0**.**54**	−0.23	−0.15	−0.03	0.18	−0.25	0.14	0.22	**0**.**32**
*intensity cues*													
IntM	Aus	**0**.**60**	**0**.**70**	**0**.**61**	**0**.**69**	0.15	0.05	**0**.**32**	**0**.**47**	0.07	**0**.**36**	0.28	**0**.**50**
	Ind	**0**.**71**	**0**.**75**	**0**.**70**	**0**.**78**	0.08	0.09	**0**.**36**	**0**.**60**	0.06	**0**.**49**	**0**.**55**	**0**.**71**
IntSD	Aus	0.00	−0.02	0.00	−0.01	0.13	0.10	0.19	0.21	0.11	0.08	0.14	0.12
	Ind	0.00	−0.14	−0.02	−0.14	−0.13	−0.13	−0.07	−0.10	−0.14	−0.16	0.04	−0.06
*spectral balance cues*													
F1amplitude	Aus	0.07	0.15	0.08	0.20	**0**.**34**	0.21	0.29	0.26	0.23	0.26	**0**.**30**	0.22
	Ind	0.11	0.15	0.11	0.18	**0**.**33**	**0**.**36**	**0**.**34**	**0**.**47**	**0**.**34**	**0**.**44**	**0**.**40**	**0**.**39**
Hammarberg	Aus	−**0**.**41**	−**0**.**58**	−**0**.**40**	−**0**.**55**	−0.11	−0.09	−**0**.**36**	−**0**.**47**	−0.15	−**0**.**38**	−**0**.**32**	−**0**.**55**
	Ind	−0.24	−0.25	−0.18	−0.25	−0.18	−0.11	−**0**.**30**	−0.29	−0.17	−**0**.**34**	−**0**.**31**	−**0**.**38**
spectral slope	Aus	0.19	0.18	0.19	0.18	−0.03	0.03	−0.03	0.14	−0.14	0.06	0.01	0.12
	Ind	**0**.**34**	0.26	**0**.**33**	0.26	−**0**.**35**	−**0**.**32**	−**0**.**30**	−0.20	−**0**.**42**	−0.27	−0.19	−0.25
spectral flux *M*	Aus	**0**.**63**	**0**.**70**	**0**.**65**	**0**.**69**	0.01	−0.12	0.18	**0**.**35**	−0.05	0.20	0.16	**0**.**40**
	Ind	**0**.**75**	**0**.**77**	**0**.**74**	**0**.**80**	0.02	0.04	**0**.**30**	**0**.**57**	−0.01	**0**.**45**	**0**.**52**	**0**.**69**
spectral flux s.d.	Aus	0.18	0.25	0.20	0.22	0.08	0.07	0.20	**0**.**30**	0.09	0.12	0.21	0.29
	Ind	−0.09	−0.21	−0.09	−0.20	−0.13	−0.11	−0.13	−0.17	−0.19	−0.25	−0.07	−0.13
*temporal cues*													
VoicedSegPerSec	Aus	0.17	0.15	0.15	0.12	−**0**.**32**	−**0**.**31**	−**0**.**30**	−0.21	−**0**.**31**	−0.18	−**0**.**39**	−0.25
	Ind	0.21	0.20	0.29	0.28	−0.22	−**0**.**33**	−0.15	−0.13	−0.15	−0.21	−0.05	−0.05

For frequency cues, the mean fundamental frequency (F0M) was positively correlated with ratings of novelty and urgency; and for intensity cues, the mean voice intensity (IntM) was positively correlated with ratings of novelty, urgency, goal conduciveness and power. Regarding spectral balance cues, correlations indicated associations between an increased proportion of high- versus low-frequency energy in the voice (e.g. as indicated by low values of the Hammarberg index) and high ratings of novelty, urgency, goal conduciveness and power. The above correlations seem consistent across speaker and listener cultures, and are also in line with previous results reported in Laukka & Elfenbein [1] for American speakers and listeners.

Intrinsic pleasantness and norm compatibility showed less consistent correlations with acoustic cues in comparison to the other appraisal dimensions. However, energy in the region of the first formant (F1A) was positively correlated with ratings of both pleasantness and norm compatibility across cultures. For pleasantness, high ratings were also correlated with slower speech rate (i.e. low values of VoicedSegPerSec). Looking at individual cues, the cue with the strongest associations was the mean spectral flux (i.e. the rate of change of the power spectrum), which showed positive correlations with novelty, urgency and norm compatibility across conditions.

Overall, the acoustic correlates of appraisal dimensions showed similar trends across listener groups, although some differences are also evident in table 2. Focusing on cases where *r* differed substantially between listener groups, we note that differences occurred mainly for ratings of norm compatibility and mean fundamental frequency (F0M), amplitude of the first formant (F1A), mean voice intensity (IntM) and spectral flux.

In addition, we note that there were some cases where acoustic correlates differed between speaker groups, but not between listener groups. For example, there was a negative correlation between fundamental frequency variability (F0SD) and ratings of novelty and urgency for speakers from Australia, whereas the same correlations were positive for speakers from India. Also, there were negative correlations between spectral slope and ratings of pleasantness and goal conduciveness for Indian speakers only, and between speech rate (VoicedSegPerSec) and ratings of power for Australian speakers only.

## 4. Discussion

Individuals from Australia and India judged vocal emotion expressions from both nations in a balanced design. In a novel approach to cross-cultural research on emotion perception, participants did not judge emotion categories, but instead were instructed to rate aspects of emotion-eliciting situations, described in terms of appraisal dimensions (i.e. novelty, intrinsic pleasantness, goal conduciveness, urgency, power and norm compatibility). Participants' ratings were first compared to predictions based on appraisal theory. Results showed that 78% of mean ratings were in the predicted direction, and the proportion of ratings that supported predictions was similar in both in-group and out-group conditions. Second, we tested for differences between the ratings of Australian and Indian participants, and observed relatively few group differences in these analyses (with only 18% of comparisons supporting differences between cultures). Finally, appraisal ratings were correlated with a variety of acoustic parameters with largely similar acoustic profiles across cultures. The implications of these findings are discussed below.

Taken together, the results from the perception experiment expand our conceptual understanding about the types of information that can be conveyed by emotion expressions across cultures. Our results replicate Laukka & Elfenbein [1] in showing that listeners were able to infer several features of emotion-eliciting situations from the non-verbal aspects of a speaker's voice, but also provide novel data showing that such appraisal inferences were relatively independent of the cultural backgrounds of listeners and speakers. Thus, cross-cultural transmission of non-verbal emotion signals is not limited to emotion categories—which have been the focus of most previous studies (e.g. [2,3])—but may also involve rudimentary representational information about the characteristics of the situation that elicited the emotional response in the speaker (see [15]).

The match between predictions and appraisal ratings was impressive, but not perfect. Overall, the combined findings from the present study and the previous study by Laukka & Elfenbein [1] lend support to appraisal researchers' efforts to link emotions with specific appraisal patterns [13,28]. However, some mismatches between ratings and predictions were consistent, and may deserve additional study. Such examples included ratings of novelty and urgency for happiness and sadness, which may indicate that listeners found it difficult to make the distinction between novelty and urgency. Additional study is needed to determine whether this difficulty is specific for happiness and sadness or if it generalizes to vocal expressions in general. Some appraisal and emotion combinations for which no predictions were available also showed consistent ratings across the two studies; e.g. low ratings of novelty and urgency for shame, low ratings of urgency for pride and low ratings of norm compatibility for fear and sadness. We suggest that data from human perception studies, such as the current one, can provide valuable input for the refinement of appraisal theory.

Previous cross-cultural studies in the categorical tradition have reported evidence for minimal universality where listeners perform better than chance when judging emotion categories conveyed by vocal expressions from unfamiliar cultures, coupled with evidence for in-group advantage where listeners perform better when judging vocal expressions from their own culture versus other cultures (e.g. [5–8,10]). We believe that the current results paint a similar picture for cross-cultural appraisal inferences. There was no measure of recognition accuracy in the present study, but we note that the proportions of mean ratings that were in the predicted direction were similar for both in-group and out-group conditions—which indicates that listeners overall made similar and systematic inferences across cultures. Directly testing for group differences showed relatively few differences between in-group and out-group conditions, but we observed that ratings of norm compatibility seemed to be a notable exception. This observation suggests that the effect of culture may be more pronounced for some appraisal dimensions than for others, and is in line with previous research that also reported that judgements of norm compatibility show larger cross-cultural variability compared to other appraisal dimensions [37]. Future cross-cultural studies could explicitly compare traditional categorical judgements and appraisal ratings of emotion expressions. Studies that aim to link effects of culture on the perception of specific appraisal dimensions to effects of culture on the recognition rates for specific emotions would be especially valuable.

From a methodological perspective, we note that instructing participants to rate emotion expressions on scales describing appraisal dimensions may be especially appropriate for cross-cultural research because it avoids using emotion words which may have slightly different meanings in different cultures and languages (e.g. [38]). The use of appraisal scales also allows researchers to assess perception of more subtle expressions, such as mild intensity affective states often encountered in everyday social interactions (e.g. [39]) and other affective states that do not fit well into traditional emotion categories. According to appraisal theories, there is a limited number of appraisal dimensions that lie at the root of all conceivable emotional responses (e.g. [12]). We correspondingly argue that perception of a limited number of appraisal dimensions from vocal expressions allows for recognition and differentiation of a wide palette of subtle affective states. Indeed, several previous studies have reported that listeners can make more detailed judgements of vocal expressions than what would be expected if they were only able to perceive a limited number of basic emotions or the arousal- and valence-levels of stimuli (e.g. [24,40,41]).

The findings on the acoustical correlates of appraisal dimensions give detailed information about which acoustic cues were associated with which appraisal ratings in both in-group and out-group conditions. Ratings on novelty and urgency—which are the appraisal dimensions that are most closely related to the level of arousal—received the largest correlations and were associated with all types of acoustic cues (i.e. frequency, intensity, spectral balance and temporal cues). We note that the correlation patterns were very similar for these two dimensions, which matches the observation that listeners found it difficult to separate between novelty and urgency in the rating task. By contrast, the smallest correlations were observed with intrinsic pleasantness, which is linked to the valence (positivity or negativity) of the voice. This observation is in line with previous studies which report that arousal has a larger effect on the voice compared to valence (e.g. [42]). Generally, the acoustic findings suggest that listeners used similar cues to infer appraisal information from voices in both in-group and out-group conditions, but we observed that norm compatibility again stood out with more variability between groups. This matches well the results from the rating task where norm compatibility ratings showed the largest proportion of cultural differences.

A number of important caveats should be noted when assessing the implications of the current results. This is the first study of cross-cultural perception of appraisal dimensions from the voice. As such, the results need to be replicated both directly and conceptually. For example, our study is limited because it only compared two cultures. Australia and India have different cultural profiles in terms of Hofstede's [18] dimensions, but it remains a possibility that comparisons of other nations—with other cultural profiles—might reveal additional effects of culture. The verbal content of our stimuli further consisted of neutral sentences spoken in English, in order to increase consistency across portrayals (see [10]). However, it remains a possibility that effects of culture might be more salient when vocal stimuli from different cultures are portrayed using different languages. More research is thus needed to investigate if the results of the current study can be generalized to other speaker and listener cultures and languages, preferably also using a wider selection of emotions, appraisal dimensions and acoustic parameters.

Our results are further limited in the sense that they are based on speech prosody portrayals only. Replication efforts could therefore extend the vocal expression materials to include, for example, non-linguistic vocalizations which may be more expressive than prosodic expressions (e.g. [40,41]), and spontaneous expressions which may be slightly but systematically different from posed expressions (e.g. [43]). It would also be worthwhile to investigate the cross-cultural consistency of appraisal perception using other expression modalities such as facial and bodily expressions, both in isolation and in combination with vocal expressions. Conceptually, one important avenue for future research is to investigate if listeners directly infer the appraisal dimensions from vocal stimuli, or if they first make a categorical judgement and then infer the appraisal based on their categorical judgement through a process of reverse engineering (e.g. [16]). Currently, both interpretations of the results remain possible. Studies that directly compare the time courses of emotion category perception and appraisal perception could be helpful for further investigations of this issue. Finally, we would encourage research that combines perceptual studies with measurement of objective features of expressions to further investigate the dimensionality of appraisal information in emotional expressions.

## Supplementary Material

Figure S1

## Supplementary Material

Figure S2

## Supplementary Material

Figure S3

## Supplementary Material

Table S1

## Data accessibility

Data and analysis code for this paper are available at the Open Science Framework (http://osf.io/bh9d7) [44].
