# Supplementary figures and images for: Emotion appraisal dimensions inferred from vocal expressions are consistent across cultures: a comparison between Australia and India
Source: R Soc Open Sci. 2017 Nov 15;4(11):170912. doi: 10.1098/rsos.170912 (PMC5717659; doi:10.1098/rsos.170912)

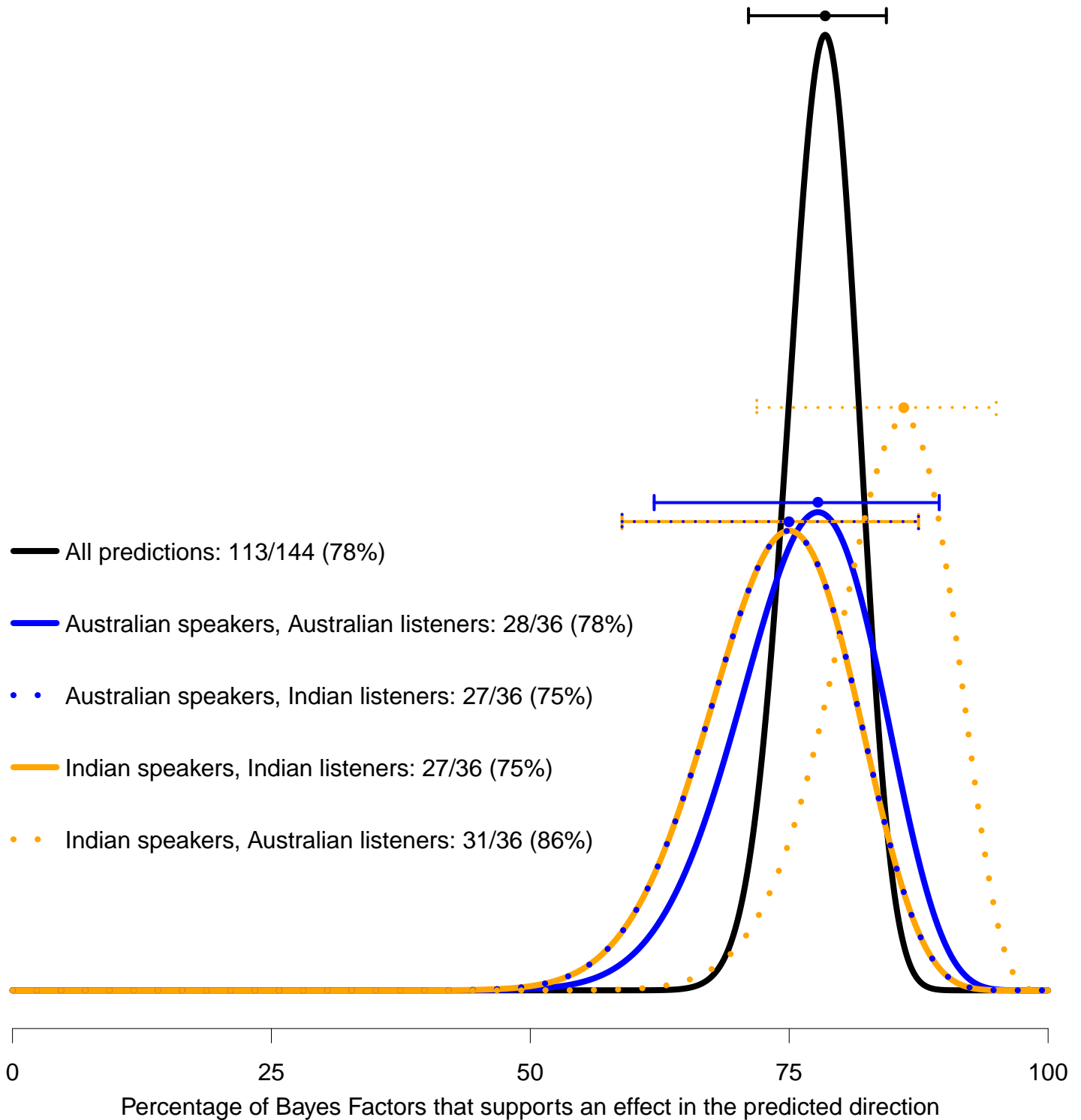

Supplement: Figure S1 [file rsos170912supp1.pdf]

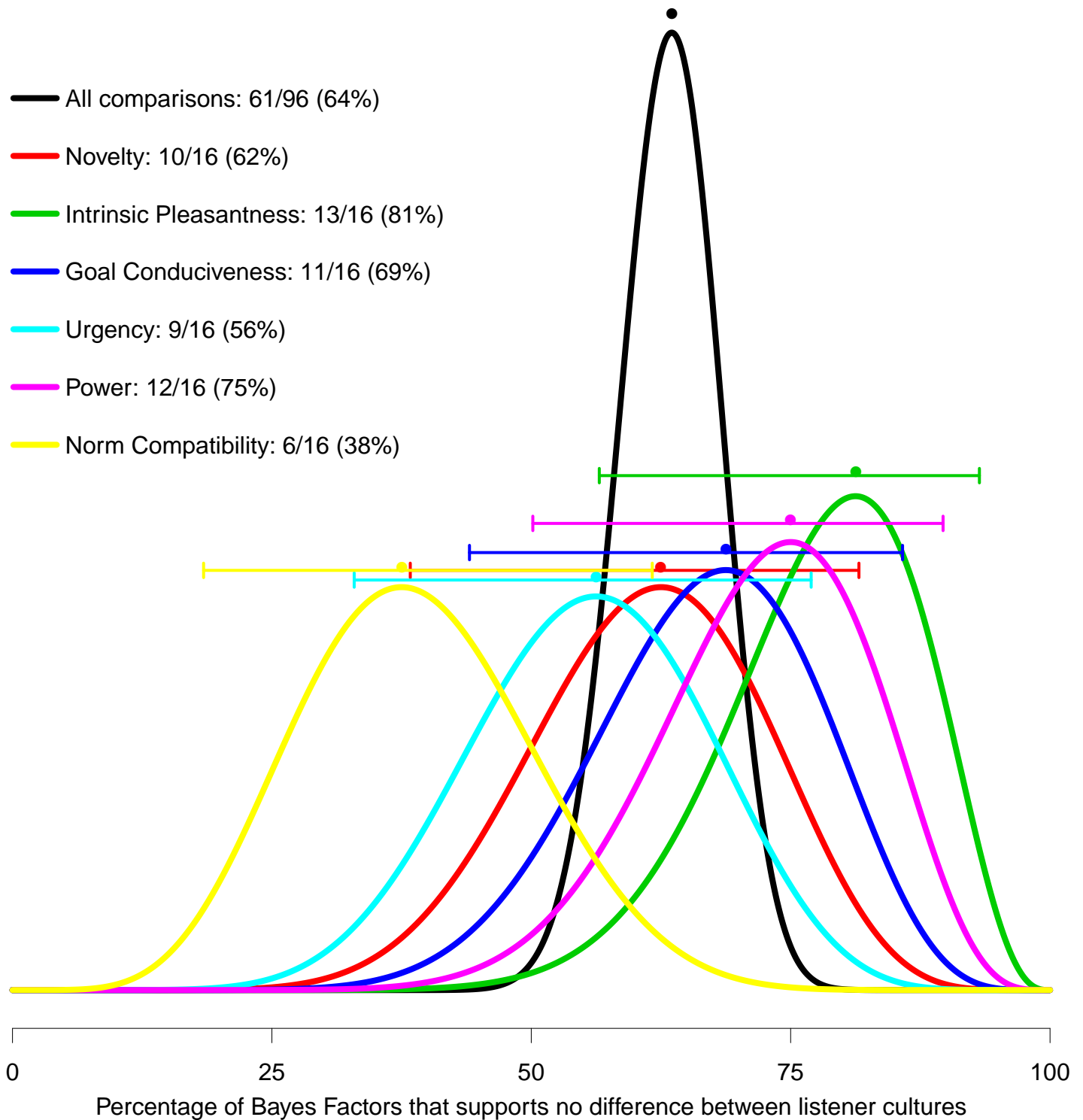

Supplement: Figure S3 [file rsos170912supp3.pdf]
