# Supplementary material for: Emotion appraisal dimensions inferred from vocal expressions are consistent across cultures: a comparison between Australia and India
Source: R Soc Open Sci. 2017 Nov 15;4(11):170912. doi: 10.1098/rsos.170912 (PMC5717659; doi:10.1098/rsos.170912)
Supplement: Figure S2 [file rsos170912supp2.pdf]

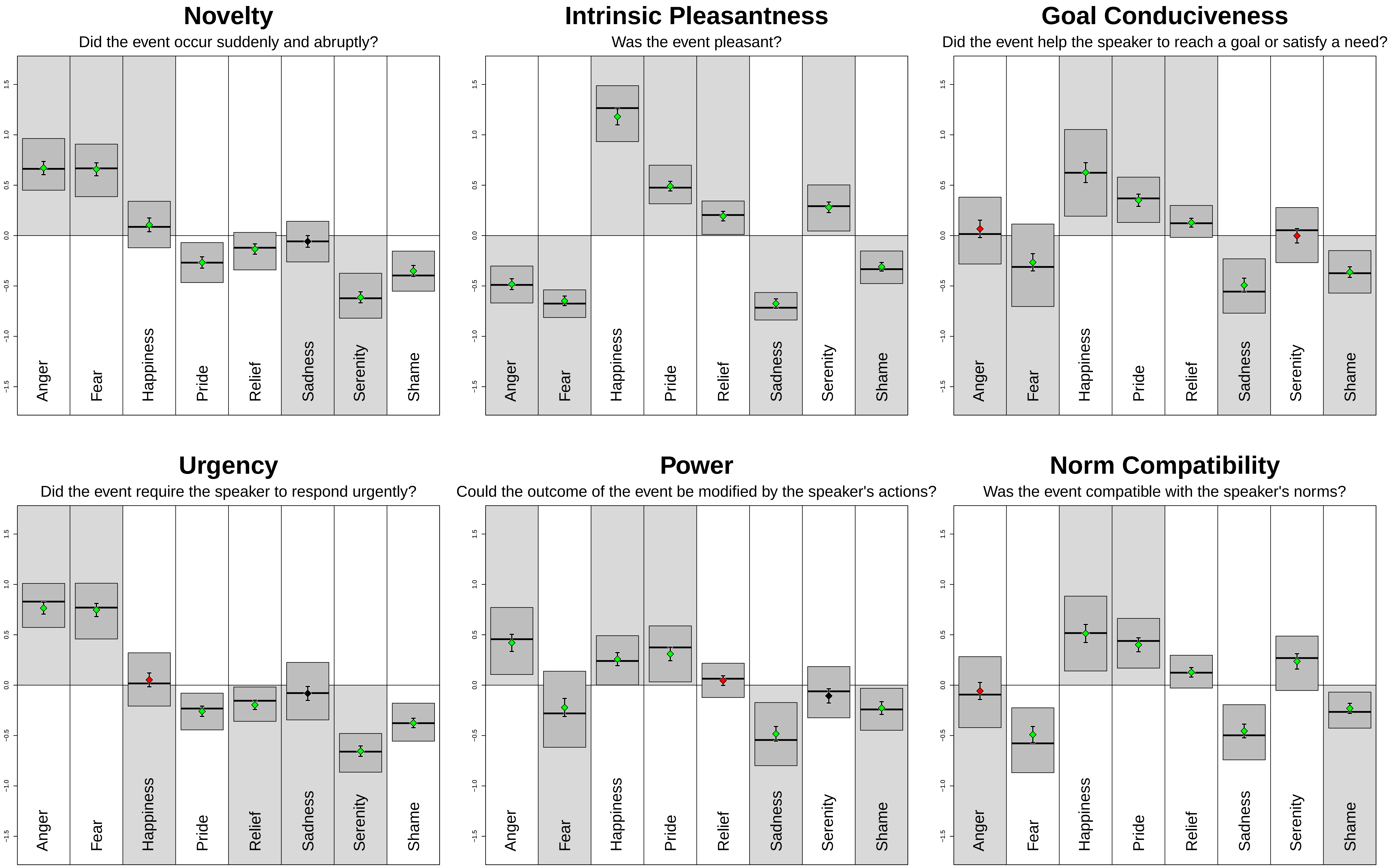
Novelty
Did the event occur suddenly and abruptly?
Intrinsic Pleasantness
Was the event pleasant?
Goal Conduciveness
Did the event help the speaker to reach a goal or satisfy a need?
Urgency
Did the event require the speaker to respond urgently?
Power
Could the outcome of the event be modified by the speaker's actions?
Norm Compatibility
Was the event compatible with the speaker's norms?
Anger
Fear
Happiness
Pride
Relief
Sadness
Serenity
Shame
1.5
1.0
0.5
0.0
-0.5
-1.0
-1.5
